# Trends in the Incidence of Lower Extremity Amputations in People with and without Diabetes over a Five-Year Period in the Republic of Ireland

**DOI:** 10.1371/journal.pone.0041492

**Published:** 2012-07-31

**Authors:** Claire M. Buckley, Anne O’Farrell, Ronan J. Canavan, Anthony D. Lynch, Davida V. De La Harpe, Colin P. Bradley, Ivan J. Perry

**Affiliations:** 1 Department of General Practice, University College Cork, Cork, Ireland; 2 Department of Epidemiology and Public Health, University College Cork, Cork, Ireland; 3 Health Intelligence, Clinical and Services Strategy, Health Service Executive (HSE), Dublin, Ireland; 4 Department of Endocrinology, St Vincent’s Hospital, Dublin, Ireland; Tehran University of Medical Sciences, Islamic Republic of Iran

## Abstract

**Aims:**

To describe trends in the incidence of non-traumatic amputations among people with and without diabetes and estimate the relative risk of an individual with diabetes undergoing a lower extremity amputation compared to an individual without diabetes in the Republic of Ireland.

**Methods:**

All adults who underwent a nontraumatic amputation during 2005 to 2009 were identified using HIPE (Hospital In-patient Enquiry) data. Participants were classified as having diabetes or not having diabetes. Incidence rates were calculated using the number of discharges for diabetes and non-diabetes related lower extremity amputations as the numerator and estimates of the resident population with and without diabetes as the denominator. Age-adjusted incidence rates were used for trend analysis.

**Results:**

Total diabetes-related amputation rates increased non-significantly during the study period; 144.2 in 2005 to 175.7 in 2009 per 100,000 people with diabetes (p = 0.11). Total non-diabetes related amputation rates dropped non-significantly from 12.0 in 2005 to 9.2 in 2009 per 100,000 people without diabetes (p = 0.16). An individual with diabetes was 22.3 (95% CI 19.1–26.1) times more likely to undergo a nontraumatic amputation than an individual without diabetes in 2005 and this did not change significantly by 2009.

**Discussion:**

This study provides the first national estimate of lower extremity amputation rates in the Republic of Ireland. Diabetes-related amputation rates have remained steady despite an increase in people with diabetes. These estimates provide a base-line and will allow follow-up over time.

## Introduction

Diabetic foot disease is a major health problem and unfortunately, Lower Extremity Amputation (LEA) remains a common outcome. LEA is a significant complication that is costly to individuals economically, socially and psychologically [1], [2]. The prevalence of diabetes is rising in the Republic of Ireland (ROI), with a projected increase to 5.9% of the population by 2020 [3]. Increased numbers of people with diabetes is expected to lead to an increased burden on the health services. A rise in the number of LEAs in people with diabetes is also anticipated [4].

Lower Extremity Amputations are an important indicator of the quality of care of diabetes in a population [5]. A recent review of the global variability in incidence of LEA in people with diabetes described a large variation in LEA rates in different communities, ranging from 46.1 to 9,600 per 10^5^ people with diabetes [6]. Recent data from England (2008) estimated LEA rates of 250 per 100,000 population with diabetes [7].

There is a lack of published data on LEA rates in the ROI. Base-line information on LEA rates in the ROI will facilitate comparison with other countries, benchmarking against best practice and tracking of potential improvements in the future [8]. Comparison of our LEA rates to those of our closest neighbours, the UK, is particularly interesting as their population is most similar to our own in the ROI in terms of socio-demographics. Thus, the objectives of this study are to determine diabetes-related and non-diabetes related LEA incidence rates and estimate the relative risk of an individual with diabetes undergoing a lower extremity amputation compared to an individual without diabetes in the ROI.

## Methods

### Ethics Statement

Aggregate data from a routine dataset was obtained via Health Atlas. Ethical approval was not required. As this data cannot be linked to individual patients, individual patient consent was not obtained. All data included is publicly available and no personal information was handled.

This study is a retrospective review of data on hospital discharges from the Hospital In-Patient Enquiry (HIPE) dataset over a 5 year period in the ROI. HIPE collects information on day and in-patient discharges from acute public hospitals including private patients treated in Health Service Executive hospitals. If a patient dies in hospital, a discharge summary is completed and the HIPE system collects this patient’s details. Data extracted for each hospital discharge includes patient age, length of hospital stay, discharge status, principal diagnosis and procedure and up to 20 secondary diagnoses and procedures. HIPE is available on Health Atlas; a mapping software system [9]. HIPE data has been previously used in other studies to identify hospital admission rates for pancreatitis and alcohol intoxication [10], [11].

The numbers of discharges for patients with diabetes (ICD-10 codes E10–E11) for any cause between 1^st^ January 2005 and 31^st^ December 2009 were initially identified. Next, all discharges for LEA procedures performed on patients between 1^st^ January 2005 and 31^st^ December 2009 were identified through an ICD-10 procedure code (ICD-10 codes 1484, 1505 and 1533) in any procedure field. LEAs were categorised as major or minor. A major LEA was defined as through or proximal to the ankle joint (ICD 10 codes 44361-00, 44361-01, 44367-01, 44367-02, 44370-00, 44373-00, 44367-00); a minor LEA as one distal to the ankle joint (ICD-10 Codes 44338-00, 44358-00, 90557-00, 44364-00, 44364-01) [7]. All traumatic LEAs, defined by any trauma-related code of the lower extremity in any diagnosis field (ICD-10 codes S70-99, T00-35, Wxx, and Xxx) were excluded from the analysis. For each discharge, diabetes status was classified as no diabetes or type 1 or type 2 diabetes (ICD-10 codes E10–E11) recorded in any diagnosis field. Other forms of diabetes (ICD-10 codes E12–E14) were excluded.

The denominators used were the total estimated resident population with and without diabetes in the ROI in each year of the study. The Institute of Public Health, Ireland provided estimates of the prevalence rate of diabetes in the population in 2007 in a study ‘Making Chronic Conditions Count: A systematic approach to estimating and forecasting estimate of the diabetic population in Ireland as population prevalence on the island of Ireland’. [3]. Population prevalence rate estimates (incorporating obesity and smoking) for 3 different age-categories for the year 2007 were calculated by the IPH; 0.6% for 20–29 years, 3.0% for 30–59 years and 13.2% for 60+ years. These age-specific 2007 estimated prevalence rates were applied to 2005–2009 population data [12]. A census took place in the ROI in 2006 and data for other study years are inter-censal estimates. The estimated population without diabetes was calculated by subtracting the estimated population with diabetes from the estimated total population for each year.

### Statistical Analysis

Statistical analyses were performed using the Stats Direct statistical package. Age-adjusted incidence rates were standardised to the EU standard population and the 95% confidence intervals were based on the Poisson distribution. Incidence rates in 3 different age-categories (20–29, 30–59 and 60+ years) were calculated. Cuzick’s trend test was used to test for significant changes over time. A two-sided p<0.05 was considered statistically significant. The relative risk of an individual with diabetes undergoing a LEA (minor, major and any) compared with that of an individual without diabetes was estimated with 95% confidence intervals.

## Results

### Number of Discharges Per Year – Diabetes-related

**Figure 1:**
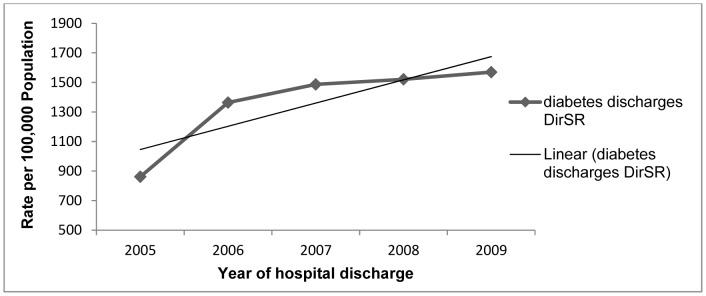
Hospital Discharge rates. Direct age standardised rate of hospital discharges of patients with diabetes for any cause, per 100,000 total population.

Figure 1 demonstrates the number of hospital discharges for any patient with diabetes increased from 35,938 discharges in 2005 to 65,473 discharges in 2009, with the age standardised rate increasing significantly from 861 to 1,569 per 100,000 *total* population (test of linear trend, p = 0.04).

### Number of Discharges Per Year – Diabetes-Related LEAs

During 2005–2009, 2,776 patients underwent non-traumatic LEA in the ROI. Of all amputations, 1654 (53.5%) occurred among people with diabetes.

The median length of stay for a LEA in people with diabetes was 24 days (range 1–539 days); with a total of 60,332 bed days occupied over the 5 year study period. The mortality rate during a hospital admission for LEA in patients with diabetes was 6.4%. The majority of patients with diabetes admitted for LEA were discharged home post-operatively (64.6%). A further 16.1% of patients were discharged to nursing homes and the remaining 12.9% were discharged elsewhere (e.g. transfer to other hospital, self-discharge etc.).

### Diabetes-related LEA Rates

**Figure 2:**
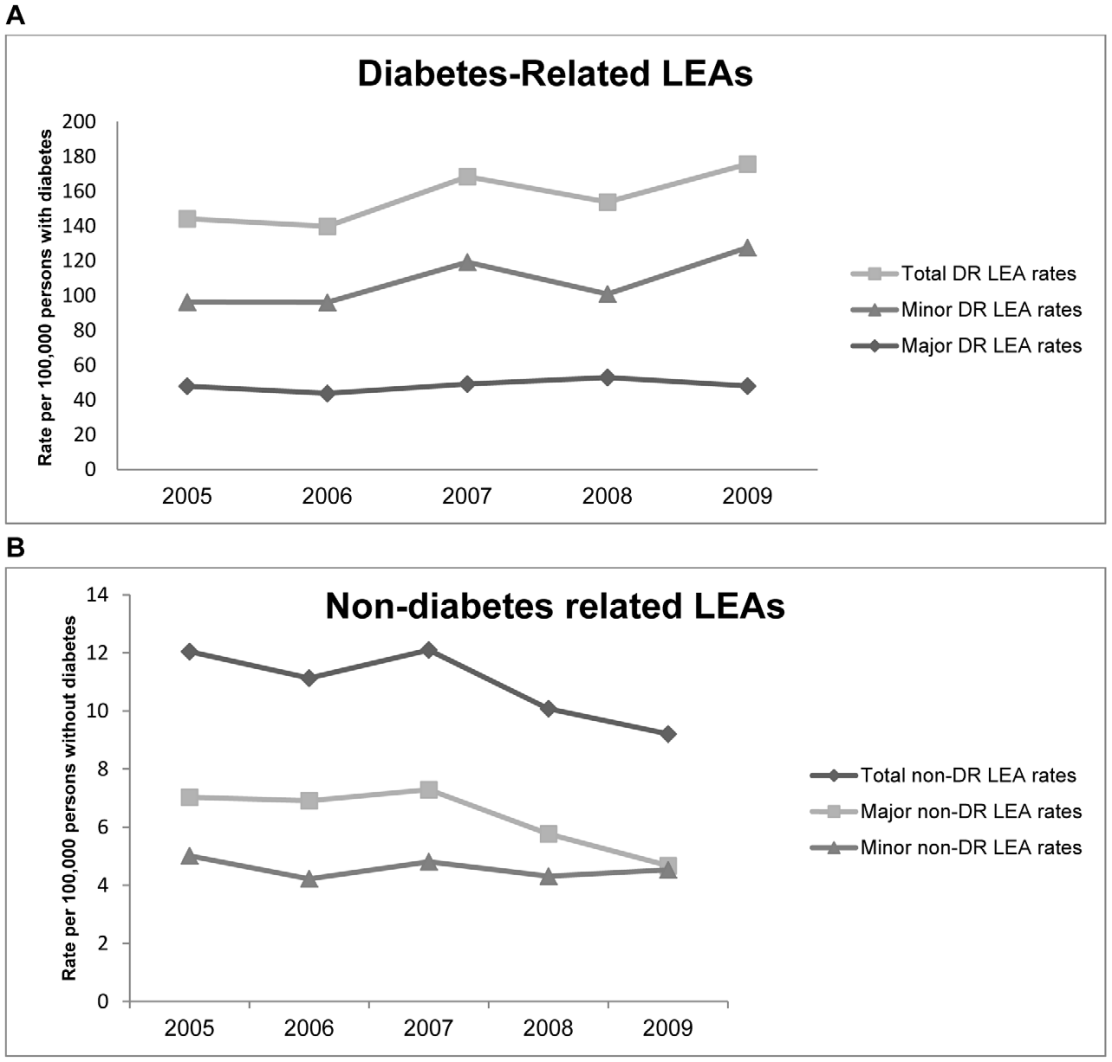
LEA Incidence rates. Changes in total, major and minor LEA incidence rates in A) people with diabetes expressed per 100,000 people with diabetes and B) people without diabetes expressed per 100,000 people without diabetes.

**Figure 3:**
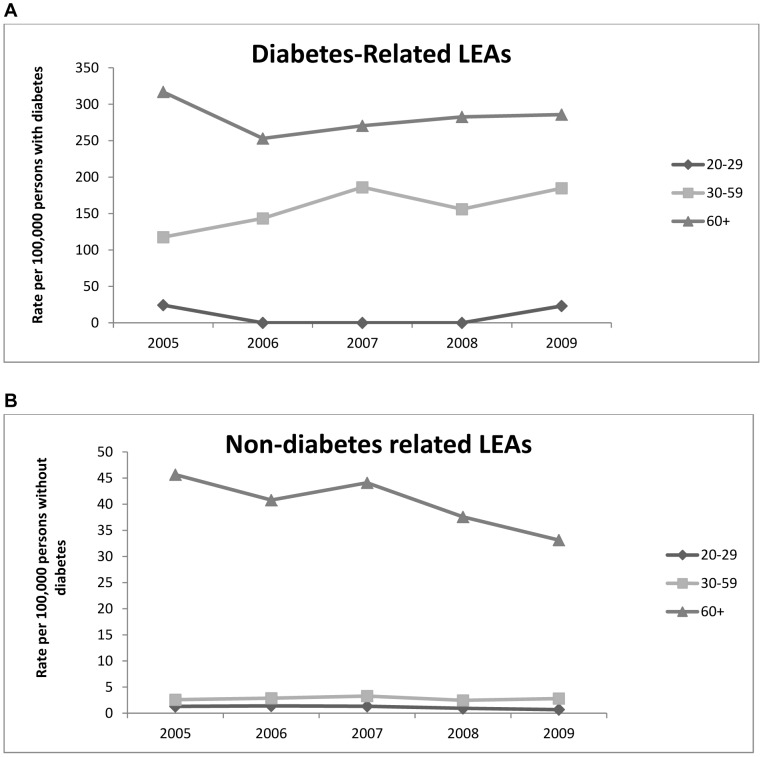
LEA Incidence rates. Changes in total LEA incidence rates in A) people with diabetes expressed per 100,000 people with diabetes and B) people without diabetes expressed per 100,000 people without diabetes, by age: 20–29 years, 30–59 years, 60+ years.

The numbers of discharges for diabetes-related LEAs used as the numerator are described in table 1. Denominator data is outlined in table 2. Figure 2A outlines the trends in diabetes-related LEA rates during 2005–2009. Total diabetes-related LEA rates increased non-significantly from 144.2(95% CI 123.2–166.9) in 2005 to 175.7(95% CI 152.3–200.9) in 2009 per 100,000 people with diabetes (p = 0.11). Major diabetes-related amputation rates remained steady during the study period; 47.9 (95% CI 37.8–59.5) to 48.0(95% CI 37.3–60.4) per 100,000 people with diabetes (p = 0.23). Minor diabetes-related amputations rates increased non-significantly from 96.2 (95% CI 78.2–116.3) to 127.6 (95% CI 107.2–150.1) per 100,000 people with diabetes (p = 0.11). Figure 3A illustrates the trends in diabetes- related LEA rates over time in 3 different age-categories. A non-significant increase was seen in the 30–59 years age-category from 117.6 (95% CI 89.5–151.7) to 184.7 (95% CI 150.5–225.5) per 100,000 people with diabetes (p = 0.16).

**Table 1:** Number of discharges for Diabetes Related-LEAs per year.

	2005	2006	2007	2008	2009
*Total DR-LEAs*	324	291	334	338	367
*Major DR-LEAs*	125	98	119	129	114
*Minor DR-LEAs*	199	193	215	209	253

**Table 2:** Estimated population with and without diabetes.

	2005	2006	2007	2008	2009
*With diabetes*	137554	141646	144442	148211	151698
*Without diabetes*	2,850,041	2,943,496	3,024,785	3,077,780	3,091,222

### Nondiabetes-related LEA Rates

The estimated population without diabetes used as the denominator to calculate nondiabetes-related LEA rates is described in table 2. There was a fall in total and major non-DRLEAs over the 5 years (Figure 2B). The total non-DR LEA rate dropped non-significantly from 12.0 (95% CI 10.7–13.5) to 9.2 (95% CI 8.1–10.4) per 100,000 people without diabetes (p = 0.16). The rate for major non-DR LEAs dropped from 7.0 (95% CI 6.0–8.2) to 4.7 (95% CI 3.9–5.6) per 100,000 people without diabetes (p = 0.16). The rate of minor non-DRLEAs remained steady changing from 5.0 (95% CI 4.2–6.0) to 4.5 (95% CI 3.8–5.4) per 100,000 people without diabetes (p = 0.55). Nondiabetes-related LEA rates remained low in the younger age-categories and a non-significant decrease from 45.7 (95% CI 40.2–51.7) to 33.1 (95% CI 28.7–38.0) per 100,000 people without diabetes was observed in the 60+ year age-category (p = 0.07) (figure 3B).

### Relative Risk of LEA

The risk of an individual with diabetes undergoing a LEA was 22.3 times that of an individual without diabetes in 2005 and did not change significantly by 2009 (p = 0.4) (see Table 3).

**Table 3:** Relative risk (95% CI) of Lower Extremity Amputations in people with diabetes compared with those without diabetes between 2005 and 2009 in the ROI.

	AllAmputations	MajorAmputations	Minoramputations
2005	22.3 (19.1–26.1)	14.8 (11.8–18.6)	32.7 (26.2–40.9)
2006	21 (17.8–24.7)	11.5 (9.0–14.7)	36.1 (28.6–45.6)
2007	21.9 (18.8–25.6)	13.0 (10.3–16.3)	35.5 (28.5–44.1)
2008	22 (18.9–25.6)	17.2 (13.6–21.7)	37.4 (29.8–46.9)
2009	29.2 (24.9–34.3)	17.9 (13.9–23.0)	40.9 (33.0–50.7)

## Discussion

Diabetes is on the rise and is impacting on health services in the ROI. This is reflected in a significant increase in the age-standardised rate of hospital discharges for all patients with diabetes during 2005–2009 per 100,000 total population.

This study determined incidence rates of diabetes and non-diabetes related LEAs in people with and without diabetes. A non-significant rise in total (major & minor) diabetes-related LEA rates from 144.2 to 175.7 per 100,000 people with diabetes was found. We are cognisant that our results should be compared to data from studies of similar methodology [13]. In England, from 2004 to 2008, Vamos et al described a decrease in total LEA rates from 275 to 250 per 100,000 population with diabetes [7]. Elsewhere in Europe, in the Canary Islands in Spain, the incidence rate was 319.7 per 100,000 people with diabetes in 2001/2002 [14]. Fosse et al reported an incidence rate of LEA of 158 per 100,000 people with diabetes in France in 2003 [15]. However, the methodology in the French study is slightly different as rates were sex and age standardised while the rates in this study from the ROI are only age standardised.

Other studies have used slightly different methods to determine the numerator but are worthy of mention. In the UK, Canavan et al described a decrease in total LEA rates, 564.3 to 176 per 100,000 population with diabetes in the South Tees from 1995–2000; while in Ipswich from 1995 to 2005, total amputations fell from 532 to 160 per 100,000 people with diabetes [16], [17].

In the Netherlands, incidence was reported as 363 per 100,000 people with diabetes in 2000 [18].

Outside Europe, among U.S. Medicare beneficiaries with diabetes, the annual incidence of total LEA was 500 in 2006, 460 in 2007, and 450 in 2008 per 100,000 people with diabetes [19]. In Texas, Lavery et al recently described an incidence of 590 per 100,000 persons with diabetes per year [20]. Direct comparison with previous studies is not possible due to methodological differences. However, notwithstanding the difference in time periods, LEA rates from the ROI seem broadly in line with the rest of the world or, perhaps, somewhat lower. The apparent difference, though, may be due to incomplete data capture or possibly, lesser representation of ethnic groups with higher risk for LEA in the Irish population [6], [21], [22].

An early minor amputation can prevent a later major amputation [23]. Thus, minor amputations may reflect improved quality of care with earlier intervention; consequently preventing the progression from minor to major amputation. For this reason and as the functional outcomes for major and minor LEAs differ markedly, it is prudent to examine major and minor LEA rates separately [24]. During the study period in the ROI, major LEA rates fluctuated between 47.9 and 48.0 per 100,000 people with diabetes while minor LEA rates rose from 96.2 to 127.6 per 100,000 people with diabetes. We can hypothesise that these trends in minor LEA rates may be a sign of earlier intervention and changes in clinical practice over time. However, it is important to emphasise that these changes were non-significant. Our findings are comparable to England, where major LEA rates dropped from 118 to 102 per 100,000 people with diabetes from 2004 to 2008 [7].

An increase in diabetes-related LEA rates in the 30–59 year old age-category was observed. Although this increase did not reach statistical significance, it is, nonetheless, worrisome (figure 3A). As the age of onset of diabetes reduces and the age of survival increases, more time exists for complications of diabetes including LEA to develop [25], [26]. The trend seen here suggests that these complications are now occurring at a younger age. However, as the event rate is low, it is difficult to provide incidence rates by age category with acceptable reliability. Fortunately, non-diabetes related LEAs are rare events in adults <60 years and rates in those 60+ years are reducing. This is consistent with improved cardiovascular health in the ROI in recent decades [27].

It is suggested that ideally, the relative risk of a person with diabetes undergoing a LEA should be similar to that of a person without diabetes [16]. Recently published data from England reports a relative risk of 21.2 for 2008–2009 [7]. Our relative risk of 29 in 2009 suggests that patients with diabetes in the ROI have a greater risk of LEA than our neighbours in the UK. There are a number of possible reasons for this; including healthcare systems in place, foot care practices and population differences.

### Strengths and Weaknesses

This study has a number of strengths; it is population based and uses methodology comparable to other countries [7], [15]. Comparing results of different studies analysing LEAs in patients with diabetes requires caution; different authors use different techniques and operational definitions to identify numerators (e.g. total versus major amputations) and denominators (e.g. total population versus population at risk) [13]. In this study, total, major and minor amputation rates are described per 100,000 people with diabetes, as expression of incidence per ‘at risk’ population is a better reflection of the true state [28], [29]. A non-diabetes comparator is also included to allow calculation of relative risk. However, limitations affecting both the numerator and denominator need to be considered.

As all LEA procedures are carried out in hospital, the numbers of discharges for LEAs serve as a proxy measure of the number of LEAs in this study and were used as the numerator. The use of this numerator to calculate LEA incidence, instead of the actual number of LEA procedures performed has been criticised [30]. Van Houtum argues that more than one LEA can occur during one admission in some patients and these extra amputation procedures are not captured. While this is indisputable, hospital discharges have been used in previous reports so it is valid to compare our results with other studies of the same methodology.

This study does not include auto-amputations (spontaneous detachments of distal limb extremities). Whether autoamputation is an optimal management strategy in patients with diabetes is the subject of debate [31]. Regardless, patients with diabetes that undergo autoamputation have been omitted from previous studies and this study also, as this data could not be captured [32].

Concerns have been raised on the accuracy of routinely recorded datasets such as HIPE [33], [34]. Reporting procedures in HIPE are generally regarded in Ireland as fairly robust. They are incentivised by links between HIPE reporting and re-imbursement mechanisms within the health system although incomplete data capture is still a possibility which necessitates some caution in the interpretation of our data. Of note, no changes in recording practices occurred during the study period. Also, divergent trends were observed between major and minor diabetes-related LEA rates in people with diabetes (figure 2). This suggests that data collection methods are robust and casts doubt on the concern that the increases noted in LEA rates in people with diabetes may be due to improved IT systems and data recording. It is noteworthy that our estimates are similar to those from the UK, where accuracy of discharge coding is documented as high, in the order of 97%, for operations [34] Furthermore, discharge databases have been used in previous studies to assess trends in LEA procedures in patients with and without diabetes [7], [23], [35]–[37].

In the denominator, the population with diabetes was estimated from prevalence rates calculated by the Institute of Public Health for 1 year, 2007, and these were applied to all years in the study (table 2). Thus, this study assumes that the prevalence rate of diabetes remained constant between 2005 and 2009. We calculated that in the ROI, the population >20 years with diabetes increased by 10% between 2005 and 2009. A Scottish study found that if stable mortality rates are assumed, then the natural increase in prevalence over a five year period is expected to be 10% if incidence does not change at all [38]. This study found a 10% increase in prevalence over five years. However, incidence is most likely rising as suggested by the significant increase in the age-standardised rates of hospital discharges for people with diabetes for any cause (figure 1). It seems likely therefore, that our estimates of the population with diabetes are conservative. This theory is compounded by recent publications which quoted a 18% increase in diabetes prevalence over 5 years in England (2004–2008) and a 65% increase over 11 years in Finland (1997–2007) [7], [37]. However, in the absence of a diabetic register, the current estimates of the actual population with diabetes are the best available in the ROI. A diabetic register would be a useful resource for gathering precise prevalence data for health planning, policy and research.

### Implications for Practice and Future Research

With optimal care, many LEAs in people with diabetes are preventable [15], [39]. Care of diabetic foot disease needs to be continuously improved to prevent future amputations. Across the globe, changes are being implemented including the introduction of structured footcare programmes and multidisciplinary teams [16_ENREF_16,17,40,41]. A national programme to provide evidence-based care of diabetes is currently being implemented in the ROI. This includes the development of additional multidisciplinary teams. Concurrently, the structure of diabetes care in the ROI is currently undergoing a transformation with the focus of care shifting from hospital to community-based management**.** This study provides the first national estimate of LEA rates which will serve as a base-line to facilitate tracking of changes and potential improvements with the new national programme and healthcare system reconfiguration.

It is re-assuring that these first Irish LEA rates are comparable to the UK, despite a disparity between countries in resources contributed to the care of the diabetic foot. It will be challenging to reduce existing LEA rates as levels of diabetes and obesity continue to rise. In addition, there are limited resources available for diabetes care in the current economic climate. Future trends in LEA rates need to be monitored as well as trends in the prevalence of diabetes.
